# Designing a new alginate-fibrinogen biomaterial composite hydrogel for wound healing

**DOI:** 10.1038/s41598-022-11282-w

**Published:** 2022-05-04

**Authors:** Marjan Soleimanpour, Samaneh Sadat Mirhaji, Samira Jafari, Hossein Derakhshankhah, Fatemeh Mamashli, Hadi Nedaei, Mohammad Reza Karimi, Hamidreza Motasadizadeh, Yousef Fatahi, Atiyeh Ghasemi, Maryam Sadat Nezamtaheri, Mohadese Khajezade, Masoumeh Teimouri, Bahram Goliaei, Cédric Delattre, Ali Akbar Saboury

**Affiliations:** 1 Institute of Biochemistry and Biophysics, University of Tehran, Tehran, Iran; 2 Pharmaceutical Sciences Research Center, Health Institute, Kermanshah University of Medical Sciences, Kermanshah, Iran; 3 Polymer Laboratory, School of Chemistry, College of Science, University of Tehran, Tehran, Iran; 4 Department of Pharmaceutical Nanotechnology, Faculty of Pharmacy, Tehran University of Medical Sciences, Tehran, Iran; 5 Department of Petroleum Microbiology, Academic Center for Education, Culture and Research (ACECR), Shahid Beheshti University, Tehran, Iran; 6 Faculty of New Sciences and Technologies, University of Tehran, Tehran, Iran; 7 Institut Universitaire de France (IUF), 1 Rue Descartes, 75005 Paris, France; 8 Université Clermont Auvergne, CNRS, Clermont Auvergne INP, Institut Pascal, 63000 Clermont-Ferrand, France

**Keywords:** Biological techniques, Biophysics, Medical research, Materials science

## Abstract

Wound healing is a complex process and rapid healing necessitates a proper micro-environment. Therefore, design and fabrication of an efficacious wound dressing is an impressive innovation in the field of wound healing. The fabricated wound dressing in this scenario was designed using a combination of the appropriate coagulating and anti-bacterial materials like fibrinogen (as coagulating agent), nisin (as anti-bacterial agent), ethylenediaminetetraacetic acid (as anti-bacterial agent), and alginate (as wound healing agent). Biophysical characterization showed that the interaction of fibrinogen and alginate was associated with minor changes in the secondary structure of the protein. Conformational studies showed that the protein was structurally stable at 42 °C, is the maximum temperature of the infected wound. The properties of the hydrogel such as swelling, mechanical resistance, nisin release, antibacterial activity, cytotoxicity, gel porosity, and blood coagulation were assessed. The results showed a slow release for the nisin during 48 h. Antibacterial studies showed an inhibitory effect on the growth of Gram-negative and Gram-positive bacteria. The hydrogel was also capable to absorb a considerable amount of water and provide oxygenation as well as incorporation of the drug into its structure due to its sufficient porosity. Scanning electron microscopy showed pore sizes of about 14–198 µm in the hydrogel. Cell viability studies indicated high biocompatibility of the hydrogel. Blood coagulation test also confirmed the effectiveness of the synthesized hydrogel in accelerating the process of blood clot formation. In vivo studies showed higher rates of wound healing, re-epithelialization, and collagen deposition. According to the findings from in vitro as well as in vivo studies, the designed hydrogel can be considered as a novel attractive wound dressing after further prerequisite assessments.

## Introduction

The skin as the largest organ in the body is able to protect the body as well as its constituents through acting as a barrier against mechanical and microbial damage^1^. Hence, skin damages or wounds are a major medical problem today^2^. A population of nearly six million people worldwide struggles with various kinds of wounds, which disrupts their lifestyles and reduces their quality of life. Particularly, chronic wounds that are severe and take more than 12 weeks to heal, are often associated with recurrence. These wounds are caused by specific diseases such as diabetes and tumor diseases as well as severe physiological infections^3^. Therefore, the design of an effective wound dressing is a major perquisite to enhance the healing rate of wounds.

A proper wound dressing should provide a favorable micro-environment for wound healing via an appropriate gas exchange with the environment, while not permeable to dust particles and germs. Low adhesion to the wound site as well as easy separation from the wound surface are other characteristics of a proper wound dressing^4^. Till now, diverse dressings for wounds have been developed, including hydrocolloids, films, foams, hydrogels, and so on. The hydrophilic nature of hydrogel dressings presents many benefits like the absorption of extra wound discharge, protecting the humid environment, penetration of oxygen to the wound site, and reducing the patient's pain^5^.

Recent studies have revealed the ability of some natural biopolymers such as, chitosan, collagen, and alginate to facilitate the wound healing process^6–10^. A complex hydrogel made of chitosan and alginate containing fibroblast growth factors/ Vascular endothelial Cadherin has been shown to accelerate skin wound regeneration^11^. Furthermore, another chitosan-alginate hydrogel composite containing nisin has been developed and shown to be effective in healing acute wounds^12^. Since the 1980s, a large number of dressings containing these two natural compounds have been synthesized and used to treat skin wounds, especially diabetic ones, which is one of the leading causes of global mortality^13^. As a highly hydrophilic carbohydrate, alginate is derived from brown sea algae and bacterial sources like *Azotobacter* and *Pseudomonas*. It is a polyanionic copolymer containing homopolymeric blocks of (1,4)-linked-*β*-D-mannuronate (M) and *α*-L-guluronate (G) residues^14,15^. One of the most important properties of alginate is its tendency toward metal ions, which helps to make very strong gels^16^. It should be noted that the formed structure is a determinant factor for trapping cells, DNA, and protein^14^. Furthermore, alginate is endowed with non-immunogenicity, chemical versatility, being cost-effective, low toxicity, biodegradability, biocompatibility, unique water absorption, and remarkable crosslinking capability; these allowed alginate to attract much attention in medical applications, especially regenerative medicine and wound healing^17–19^.

On the other hand, one of the important events in the wound healing process is the rapid coagulation of blood at the wound site in which fibrinogen plays an important role. Fibrinogen is a large (340 kDa) glycoprotein, approximately 45 nm in length, consisting of three pairs of polypeptide chains (α2β2γ2) connected through five disulfide bridges in the *N*-terminal domain. In sum, 29 intra- and inter-chain disulfide bonds stabilize the protein^20,21^. Fibrinogen along with thrombin are important factors in blood coagulation, cellular attachment, inflammatory responses, and wound healing^22^. Recently, self-assembled fibrinogen scaffolds have been developed with the potential application in wound healing systems^23^.

The other important factor that should be considered in the wound healing process is bacterial infections, which have been a great medical challenge today due to the emergence of antibiotic resistance. Nisin is an antibacterial peptide with a molecular weight of 3.3 kDa and 34 amino acids, belongs to the group A Lantibiotics, and acts against Gram-positive bacteria^24,25^. Nisin has many applications in food preservation and medicine, including wound healing; it acts against some species of bacteria such as *Staphylococcus aureus* and *Clostridium difficile*^26,27^. The mechanism of the antibacterial effect of nisin is through forming pores in the bacterial cell wall. Although nisin acts against a wide range of Gram-positive bacteria, it does not affect Gram-negative bacteria and fungi^28^. It is well documented that nisin presents antibacterial effects against gram-negative strains in the presence of ethylene diamine tetraacetic acid (EDTA)^25^.

Regarding the above-mentioned issues, we designed a wound dressing hydrogel composed of alginate, fibrinogen, nisin, and EDTA for the repair of skin ulcers. We started by studying the interaction between fibrinogen and alginate, the main ingredient of the hydrogel, using spectroscopic techniques. Then, we prepared and optimized the hydrogel and characterized it using various methodologies. Finally, we performed in vivo studies and tested the prepared hydrogel wound dressing on the ulcers made on rats and observed improvements in the healing process.

**Figure 1:**
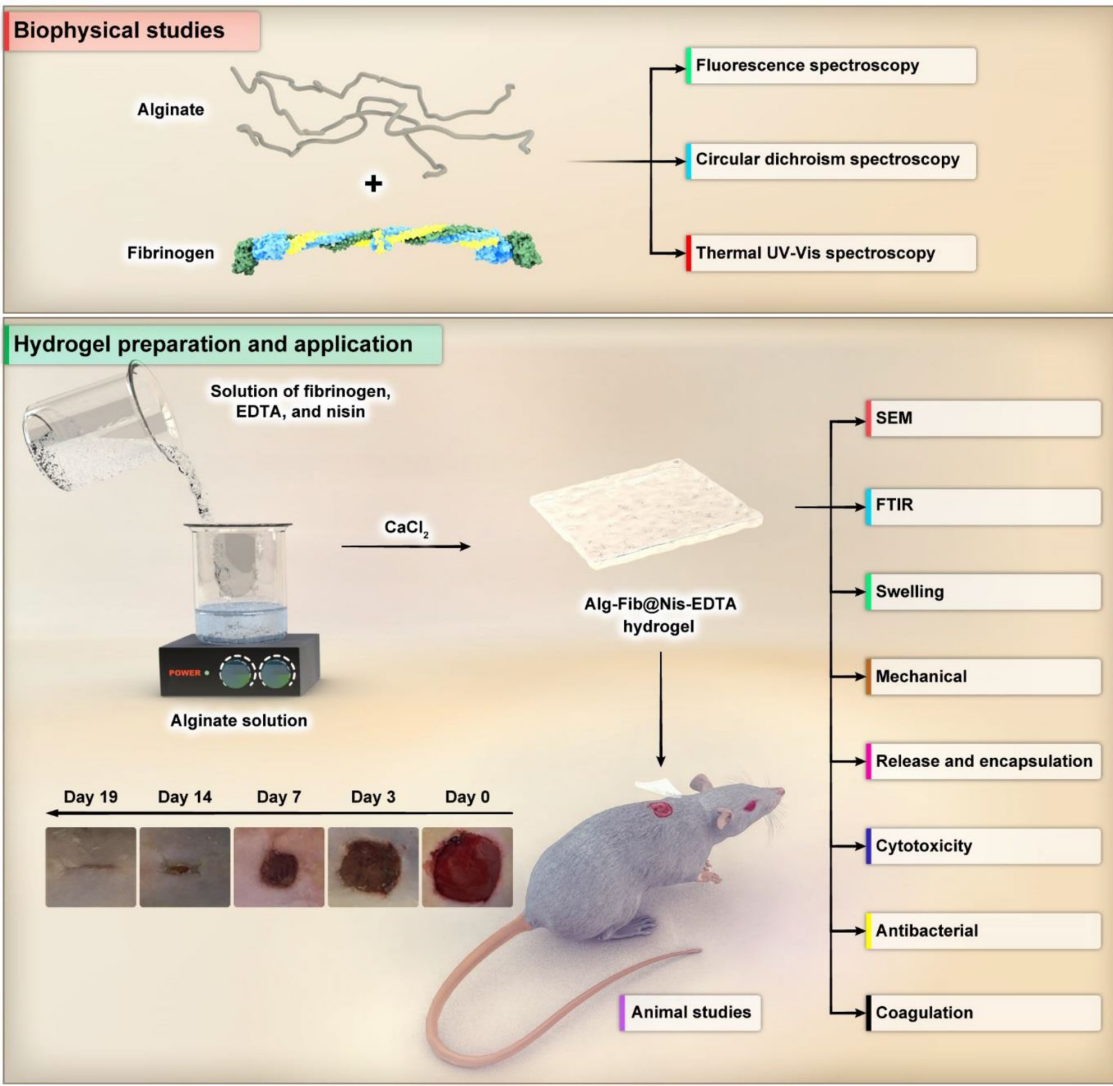
The schematic diagram of material preparation and application shows an overview of the present study. In the first step, biophysical and structural studies on fibrinogen protein in the presence and absence of alginate polymer were performed using fluorescence spectroscopy, Circular dichroism spectroscopy and Thermal UV–Vis spectroscopy. In the second step, the hydrogel was prepared and characterized through visual evaluation and scanning electron microscopy, Fourier-transform infrared spectroscopy, swelling ratio, mechanical study, in vitro nisin release, encapsulation efficiency**,** cytotoxicity assay**,** antibacterial activity assay, and blood coagulation assay. Finally, animal studies were performed. The optimized hydrogel was placed on rats skins wound, and the wound healing process was observed over 19 days. (Designed by Photoshop CC 2022; http://www.adobe.com/products/photoshop.html).

The designed wound dressing in the current project is composed of the efficient agents to accelerate the wound healing process like nisin (antibacterial agent), fibrinogen (coagulating agent), calcium alginate (improving angiogenesis and collagen formation), and EDTA (antibacterial agent). Indeed, presence of the mentioned materials can provide an appropriate condition for rapid and officious wound therapy. To the best of our knowledge, such a structure has not been reported as a wound dressing. It is noteworthy to consider that the presence of several antibacterial agents (e.g. nisin and EDTA) can create synergistic antibacterial activity against gram-positive and negative bacteria. On the other hand, in current study, biophysical and structural studies were performed to investigate interactions between components of the synthesized hydrogel at the molecular level with the purpose of designing an appropriate wound dressing. In the schematic diagram shown in Fig. 1, all the study materials and phases in this research are presented.

## Experimental

### Materials

Alginate with average molecular weights of about 46 kDa (CAS: 9005-38-3), ethylenediaminetetraacetic acid (EDTA), 3-(4,5-dimethylthiazol-2-yl)-2,5-diphenyltetrazolium bromide (MTT), fibrinogen (CAS: 9001-32-5), bovine serum albumin (BSA), and nisin (CAS: 1414-45-5) were purchased from Sigma-Aldrich. NaCl, KCl, Na_2_HPO_4_, KH_2_PO_4_, CaCl_2_, Tris methylamine, and formaldehyde solution were obtained from Merck. Dulbecco's modified eagles’ medium (DMEM), fetal bovine serum (FBS), and trypsin were from Gibco. In the current study, alginate and nisin were dissolved in double distilled water, fibrinogen was dissolved in sodium phosphate buffer.

### Biophysical studies

#### Fluorescence spectroscopy

The effect of alginate polymer on fibrinogen intrinsic fluorescence was evaluated using a Varian cary eclipse spectrofluorometer at an excitation wavelength of 280 nm and emission wavelengths of 300–400 nm. During these studies, fluorescence intensity of 0.06 µM fibrinogen dissolved in 20 mM sodium phosphate buffer was measured in the presence of increasing concentrations of alginate at 25, 30, and 35 °C. The mixture of protein and alginate was vortexed for 10 s and stored at room temperature for 2 min prior to measurements^29^.

#### Circular dichroism spectroscopy

The secondary structure of 0.72 µM fibrinogen was evaluated in presence and absence of various concentrations of alginate by obtaining far UV (195–260 nm) circular dichroism (CD) spectra using an Aviv 215 Spectropolarimeter (Lakewood, New Jersey, USA) at 25 °C. In this case, fibrinogen was incubated with the mentioned concentrations of alginate for 10 min at room temperature prior to CD measurements. Quartz cells of 1 mm path length were employed for far UV CD spectroscopy. Buffer correction, smoothing, and conversion to the molar ellipticity of CD data were performed using CDSD software. Corresponding controls containing the applied alginate concentrations were prepared to correct for the buffer. Furthermore, CDNN software was applied to deconvolute the data and obtain the contents of various secondary structure elements^30,31^.

#### Thermal UV − Vis spectroscopy

Thermal stability of 13.2 µM fibrinogen in the presence and absence of 1.6 mM alginate (proportional to the ratio of protein to ligand concentration in the synthesized hydrogel) was studied by UV–visible spectroscopy. Fibrinogen absorption at 280 nm was measured using Cary 100 Bio (Varian, Australia) UV–Vis spectrophotometer. The temperature was programmed to increase 2 °C per min from 25 to 60 °C followed by 1 °C per min from 60 to 90 °C. Finally, thermal denaturation curves of fibrinogen in the presence and absence of various concentrations of alginate were prepared^32^.

### Hydrogels synthesis and characterization

#### Preparation of hydrogels

A 4.0% (w/v) stock solution of alginate was prepared in double distilled water by stirring until a homogenous solution was obtained, in which a solution composed of fibrinogen, EDTA, and nisin were added. Then, 150 mM sterile CaCl_2_ was added and vortexed to initiate gelation. The now called Alg-Fib@Nis-EDTA hydrogel preparations were provided based on varying concentrations of nisin (0.03, 0.08, and 0.15 mM). However, final concentrations of alginate (0.87 mM), fibrinogen (7.17 μM), and EDTA (50 mM) were constant in the three hydrogel preparations. We refer to the hydrogel containing only cross linked alginate as “Alg hydrogel”, and the hydrogel containing alginate, fibrinogen, nisin, and EDTA as “Alg-Fib@Nis-EDTA hydrogel” throughout the manuscript. It is worth mentioning that nisin, fibrinogen, EDTA and CaCl_2_ solutions employed in preparation of the gels were filtered through 0.2 µm sterile filters.

#### Visual evaluation and scanning electron microscopy

First, the prepared hydrogels were freeze-dried. This process was performed at a temperature of − 50 °C and a pressure of 1 mbar for 24 h to dry the hydrogels. Then, the freeze-dried hydrogel was coated with a layer of gold prior to analyze with a TE-SCAN field emission scanning electron microscope (FE-SEM), which was operated at 20 kV. The pore sizes were determined by ImageJ (version 1.53a) software^33^.

#### Fourier-transform infrared spectroscopy

Fourier-transform infrared spectroscopy (FT-IR) was performed on an FT-IR spectrophotometer (AVATAR, range from 4000 to 400 cm^−1^, Thermo, USA) with resolution of 2 cm^−1^ using KBr pellets^34^.

#### Swelling ratio

This test was performed to determine the maximum percentage of hydrogel water absorption in simulated wound fluid (SWF). The SWF contained 2.0% BSA, 0.02 M CaCl_2_, 0.4 M NaCl, and 0.08 M Tris buffer in deionized water (pH 7.5). The weight changes of the Alg-Fib@Nis-EDTA hydrogel were measured every 20 min for a period of 240 min. The swollen hydrogel was carefully removed every 20 min and placed on a filter paper to eliminate the surrounding water, and then weighed. The experiments were performed at three temperatures (25, 37 and 42 °C) each as triplicates. Swelling ratio (%) was calculated using the following equation (Eq. 1):

1 $${\text{Swelling}}\;{\text{ratio }}\left( \% \right) = \frac{{W_{s} - W_{d} }}{{W_{d} }} \times 100$$

where *W*_d_ and *W*_s_ represent the weights of the hydrogel before and after the hydration, respectively^35^.

#### Mechanical test

The freeze-dried hydrogels were initially immersed in PBS to become gel-like. Then, the uniaxial compression tests were conducted to investigate the mechanical properties at 5 mm/min rate. The compression tests were executed using cylindrical specimens with initial diameter of 15 mm and initial length of 10 mm using a SANTAM™ universal testing machine. The experiments were performed on swollen hydrogel at three temperatures (25, 37, and 42 °C) each as triplicates^36^.

#### In vitro nisin release

The release of nisin from the Alg-Fib@Nis-EDTA hydrogel was calculated by measuring its absorbance at 212 nm, followed by determining its concentration using a standard curve. The release percentage was calculated using the following equation (Eq. 2):

2 $${\text{Nisin}}\;{\text{release}}\left( {\text{\% }} \right) = {\text{released}}\;{\text{Nisin}}/{\text{total}}\;{\text{Nisin}} \times 100$$

where the “released Nisin” is equal to the amount of nisin present in the total volume of solution at different time intervals and the “total Nisin” is equal to the initial amount of nisin loaded into the hydrogel. Furthermore, release of the free nisin (not incorporated in the hydrogel) was also determined as a control. Release of free nisin was measured by adding a solution of free nisin at the same concentration as nisin loaded hydrogel into a dialysis bag and placing it in phosphate buffer followed by determining the release of free nisin during 48 h. All the experiments were carried out in triplicates^34^.

#### Encapsulation efficiency

In order to measure the efficiency of nisin encapsulation, 50 mg of Alg-Fib@Nis-EDTA hydrogel was immersed in 50 mL PBS (pH 7.4) and stirred for 30 min. It is then stirred again for 4 h with the help of a magnet at 1000 rpm. After centrifuging at 4000 rpm for 30 min, the absorption of the supernatant was analyzed at 212 nm. All the experiments were performed as triplicates. The encapsulation percentage was calculated using the following equation^34^ (Eq. 3).

3 $${\text{Encapsulation}}\;{\text{efficiency}}\left( {\text{\% }} \right) = \left( {{\text{Total}}\;{\text{amount}}\;{\text{of}}\;{\text{loaded}}\;{\text{nisin}}/{\text{Initial}}\;{\text{amount}}\;{\text{of}}\;{\text{nisin}}} \right) \times 100$$

#### Cytotoxicity assay

A431 human epidermis cell line was purchased from the National Cell Bank of Iran (Pasteur Institute, Iran). The cells were cultured in DMEM supplemented with 10% heat inactivated fetal bovine serum (FBS) and 1.0% solution of penicillin and streptomycin. The cells were cultured as a monolayer and incubated at 37 °C with 5.0% CO_2_ and 95% humidified atmosphere. A431 cells were sub-cultured using 0.25% (w/v) trypsin along with 0.03% (w/v) EDTA in PBS in order to detach cells.

Cytotoxicity of the hydrogels was examined using MTT assay. A431 cells were cultured at 20,000 cells/cm^2^ in 96 well plates and incubated for 24 h prior to treatments. Meanwhile, the hydrogel extract was prepared with 1.0 mg of hydrogels containing 0.03, 0.08, and 0.15 mM nisin in 1 mL of DMEM at 37 °C for 24 h. Then, the culture medium was aspirated, and 100 μL hydrogel extract with different concentrations of nisin was added to each well. Following 24 and 48 h incubation, the media was removed and the cells were incubated with fresh media contained 0.5 mg/ml MTT for 4 h followed by removing the media and adding 200 µl DMSO along with 25 µl Sorensen’s Glycine buffer (containing glycine, NaCl, and KCl). Using a BioTek Elisa reader, the absorbance at 570 nm was measured followed by calculation of cell viability based on the following equation^37^ (Eq. 4):

4 $${\text{Viable}}\;{\text{cells}}\,\left( \% \right) = \left( {{\text{Abs}}\;{\text{of}}\;{\text{sample}}/{\text{Abs}}\;{\text{of}}\;{\text{control}}} \right) \times 100$$

#### Antibacterial activity assay

##### Disk diffusion antibacterial susceptibility test

Plates containing LB agar culture medium with a concentration of 37 mg/L were prepared followed by seeding gram-positive *Staphylococcus aureus* (ATCC 25923) and gram-negative *Escherichia coli* (ATCC 25922). Then, sterile commercially available discs impregnated with Alg-Fib@Nis-EDTA and Alg-Fib@Nis (without EDTA) hydrogel samples while containing various concentrations of nisin along with Alg-Fib hydrogel without nisin and EDTA as control were placed on the agar plates and incubated overnight at 37 °C. Also, to evaluate the effect of crosslinking on the antibacterial properties of the synthesized hydrogel, the same samples were also tested after addition of CaCl_2_ as the crosslinking agent and gel formation. Accordingly, after preparing the gel, the gels were cut into the same size of antibacterial discs and were placed on the agar plates and incubated overnight at 37 °C. Finally, the diameter of inhibition zone around the discs and the gels were measured^38^.

##### Minimal inhibitory concentration and minimal bactericidal concentration tests

The minimal inhibitory concentration (MIC) and minimal bactericidal concentration (MBC) of nisin on *Staphylococcus aureus* (ATCC 25923) and *Escherichia coli* (ATCC 25922) were determined. Pure cultures of the bacteria were prepared in Müller-Hinton agar. One colony of each culture was transferred to the Müller-Hinton broth at a concentration of 1.0 × 10^8^ cfu/ml according to the McFarland standard followed by seeding the bacteria in 96-well plates (each well contained 100 µl of microbial stock). Quality control analysis was performed to confirm the number of bacteria used. Then, the bacteria were treated with the Alg-Fib@Nis-EDTA hydrogels (containing 0.03, 0.08, and 0.15 mM nisin), 7.17 μM fibrinogen alone, and 50 mM EDTA alone. Negative and positive control wells containing only the culture medium (without microbial or drug stocks) and the microbial stock along with culture medium, respectively, were also included. Following incubation at 37 °C for 16 h, bacterial growth was monitored by recording the OD_600_ using a UV–Vis spectrophotometer and the negative control as a blank^39,40^.

#### Blood coagulation assay

In order to examine the participation of fibrinogen present in the prepared hydrogels in blood clotting, blood coagulation test was performed. Three wound dressing samples with dimensions of 3.5 × 3.5 cm (including gauze, Alg hydrogel, and Alg-Fib@Nis-EDTA hydrogel) were each placed in a culture dish and incubated at 37 °C in a thermoshaker followed by adding 200 μl of blood. Then, 20 μl of 0.2 M CaCl_2_ was added to each sample to start coagulation. Following shaking of the samples for 10 min at 37 °C and 30 rpm, 25 ml of deionized water was added to each sample to hemolyze the red blood cells, which were not involved in the clotting process. The resulting solution was examined spectroscopically by recording the adsorption at 540 nm. The experiment was repeated three times for each sample^41^.

### Animal studies

#### In vivo wound healing test

Animal studies were performed after receiving approval from the ethics committee of Tehran University of Medical Sciences (ethical code IR.UT.SCIENCE.REC.1400.003). Effectiveness of the hydrogel dressing was determined in the Wistar rat model weighing 200–250 g. Total number of experimental rats were 36, which were divided into 3 groups of 12. The groups included: control, Alg hydrogel, and Alg-Fib@Nis-EDTA hydrogel. All the rats were kept in the standard conditions in similar cages and fed with rat food and water. At first, rats were anesthetized in a ratio of 80:20 by intraperitoneal injection of ketamine hydrochloride (50 mg/kg, ChemiDarur, Iran): xylosine (5 mg/kg ChemiDarur, Iran) followed by shaving the hair on the back of the rats using a razor and creating a 15 mm full-thickness wound of 3 cm × 3 cm with sterile surgical scalpels. The incisions were created in the dorsal lumbar region, 1.5 mm from midline on the back of rats. Finally, the rats were divided into three separate groups and the wounds of each group were covered with a special dressing for that group (3.5 cm × 3.5 cm and 2 mm thick) and fixed with sterile gauze and elastic adhesive bandage. The control wounds were only covered with sterile gauze and elastic adhesive bandage. The rats were placed in separate cages. Wound size was photographed for the first, third, seventh and fourteenth days and was measured using ImageJ software. The wound reduction was calculated according to the following equation (Eq. 5):

5 $$Wound\;reduction = \left( {\left( {A_{0} - A_{t} } \right)/A_{0} } \right) \times 100{\text{\% }}$$

where *A*_0_ is the original wound area, *A*_t_ is the wound area after time interval *t*^42^.

#### Histopathology

In order to study histopathology of the wounds, three rats of each group were selected on the 3rd, 7th, 14th, and 19th days of the experiment. The rats were euthanized using 300 mg/kg body weight of ketamine along with 20 mg/kg body weight of xylazine. Heart rate, mydriasis, and respiration were examined 15 min after injection to confirm the deaths of the animals. Then, the skin tissues were harvested and immediately fixed in the 10% neutral buffered formalin (pH 7.26) for 48 h. The fixed tissue samples were processed, embedded in paraffin, and sectioned to 5 mm thickness. Finally, the sections were stained by haematoxylin and eosin (H&E) and Masson’s trichrome (MT). The histological slides were evaluated by an independent reviewer, using light microscopy (Olympus BX51; Olympus, Tokyo, Japan). Epithelialization and fibroplasia were assessed in different groups, comparatively. Epithelialization in day 19 was assessed semiquantitatively on 3 microscopic slides in each group: 0 (without new epithelialization), 1 (25%), 2 (50%), 3 (75%), and 4 (100%). For these parameters, the results were validated by comparative analysis of an independent observer blinded to the treatment groups. Regarding collagen content and related quantification method, magnification of ×200 was employed for evaluating the samples for this criterion and the calculation was repeated for three microscopic slides^43^.

### Statistical analysis

All results were compared using Kruskal Wallis analysis for non-parametric and One way ANOVA (Tukey posthoc) for parametric data. Results with *p* values of less than 0.05 were considered statistically significant. Statistical analyses were performed using the SPSS software, version 20.0 (SPSS, Inc, Chicago, USA).

### Ethics statement

For the animal studies, all methods are performed according to the guidelines approved by Tehran University of Medical Sciences. The current project has succeeded in receiving the code of ethics with the number (IR.UT.SCIENCE.REC.1400.003) from Tehran University of Medical Sciences. All the ethical issues have been monitored during the project in accordance with the existing protocols. This study was carried out in accordance with ARRIVE guidelines (https://arriveguidelines.org).

## Results and discussion

### Interaction between fibrinogen and alginate

#### Fluorescence spectroscopy

The interaction between alginate and fibrinogen was first studied using fluorescence spectroscopy based on fibrinogen intrinsic fluorescence at three different temperatures of 25, 30 and 35 °C. According to the results presented in Fig. S1, the fluorescence intensity decreased significantly with increasing alginate concentration in the three examined temperatures. In order to understand the binding mechanism of alginate to fibrinogen, fluorescence intensity data were analyzed using the Stern–Volmer equation^44^ (Eq. 6).

6 $$\frac{{F_{0} }}{F} = 1 + K_{sv} \left[ Q \right]$$

where *F*_0_ and *F* are the fluorescence intensities of fibrinogen in the absence and presence of alginate, respectively; [*Q*] is the concentration of alginate, and *K*_sv_ is the Stern–Volmer quenching constant. Figures S2 and S3 also show *F*_0_/*F* plotted versus [*Q*] and log (*F*_0_–*F*)/*F* plotted versus log [*Q*], respectively. According to Table S1, *K*_sv_ values decrease with increasing temperature, which indicates that the quenching mechanism of fibrinogen by alginate is a static quenching. Furthermore, the binding constant (*K*_b_) and the number of binding sites (*n*) of fibrinogen for alginate were calculated using the following equation^44^ (Eq. 7).

7 $$\log \left[ {\frac{{F_{0} - F}}{F}} \right] = \log K_{b} + n\log \left[ Q \right]$$

Accordingly, the value of *n* was obtained to be approximately 1, which reveals nearly one binding site for alginate on the fibrinogen. Moreover, the binding constant showed a decreasing trend upon increasing the temperature, which can demonstrate that the binding strength of the polymer and the protein decreases with increasing temperature. The standard thermodynamic parameters such as enthalpy changes (∆H°), entropy changes (∆S°), and Gibbs free energy changes (∆G°) of the interaction between fibrinogen and alginate were obtained using the following equations^41^ (Eqs. 8, 9):

8 $$\ln K = - \Delta {\text{H}}^\circ /{\text{RT}} + \Delta {\text{S}}^\circ /{\text{R}}$$

9 $$\Delta {\text{G}}^\circ = \Delta {\text{H}}^\circ - {\text{T}}\Delta {\text{S}}^\circ = - {\text{RT}}\,\ln \,K_{b}$$

Based on the recorded data in Table S1, the values of ∆H° and ∆S° were both < 0 implying that the interaction between alginate and fibrinogen is driven by Van der Waals and hydrogen bonding forces^45^.

#### Circular dichroism spectroscopy

Far-UV circular dichroism spectroscopy (200–260 nm wavelength) was used to study the effect of 0.4–1.3 μM alginate on fibrinogen secondary structure (Fig. S4). Table S2 presents the content of secondary structure elements as obtained using CDNN software. Based on the results, no obvious change was detected in the secondary structure of fibrinogen in the presence of alginate except for a minor increase in the content of α-helices, which was accompanied by a slight reduction in the content of random coil^46^.

#### Thermal UV–Vis spectroscopy

Thermal stability of fibrinogen was studied in the presence of alginate ligand using UV–vis spectroscopy. Based on the data from the ultraviolet–visible spectrophotometry (Fig. S5), it was found that fibrinogen remained conformationally stable up to 42 °C, which is the maximum temperature of the chronic wound ^47–49^. Since the first melting temperature (T_m_) of the protein begins from about 59 °C, it is expected that the protein maintains its conformational stability when placed in the wound site.

### Hydrogel characterization

#### Visual evaluation and scanning electron microscopy

**Figure 2:**
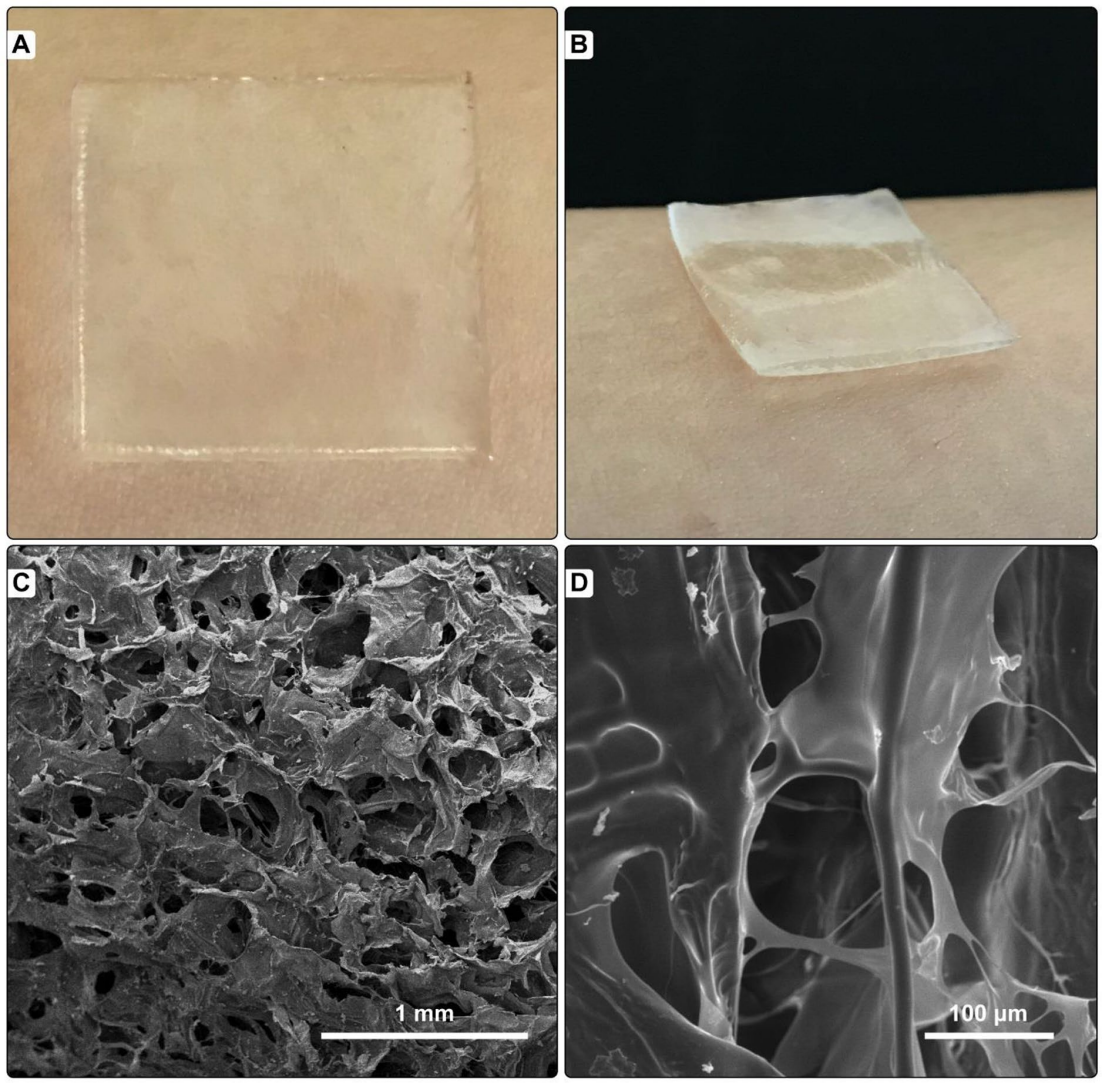
(**A**) and (**B**) The uniform, soft, and semi-opaque Alg-Fib@Nis-EDTA hydrogel. (**C**) and (**D**) Scanning electron microscopy of Alg-Fib@Nis-EDTA hydrogel with (**C**) 1 mm and (**D**) 100 µm scale bars.

The nisin containing alginate-fibrinogen (Alg-Fib@Nis-EDTA) hydrogel cross-linked with CaCl_2_ was uniform, soft, and semi-opaque. The hydrogel was easily removed from the glass plate and showed smooth surface. Figure 2A,B displays an example of the prepared hydrogel.

The morphology of alginate-fibrinogen hydrogel was studied using scanning electron microscopy (SEM) (Fig. 2C,D). The SEM image revealed a highly porous structure, rough surface, and large irregular wrinkles for the hydrogel, which presented a good agreement with published reports on the prepared hydrogels for wound dressings^37^.The diameters of the pores were measured in the range of 14–198 μm using Image J software. This pore size range is suitable for cell attachment, based on the previous studies^50–52^.

#### Fourier-transform infrared spectroscopy

**Figure 3:**
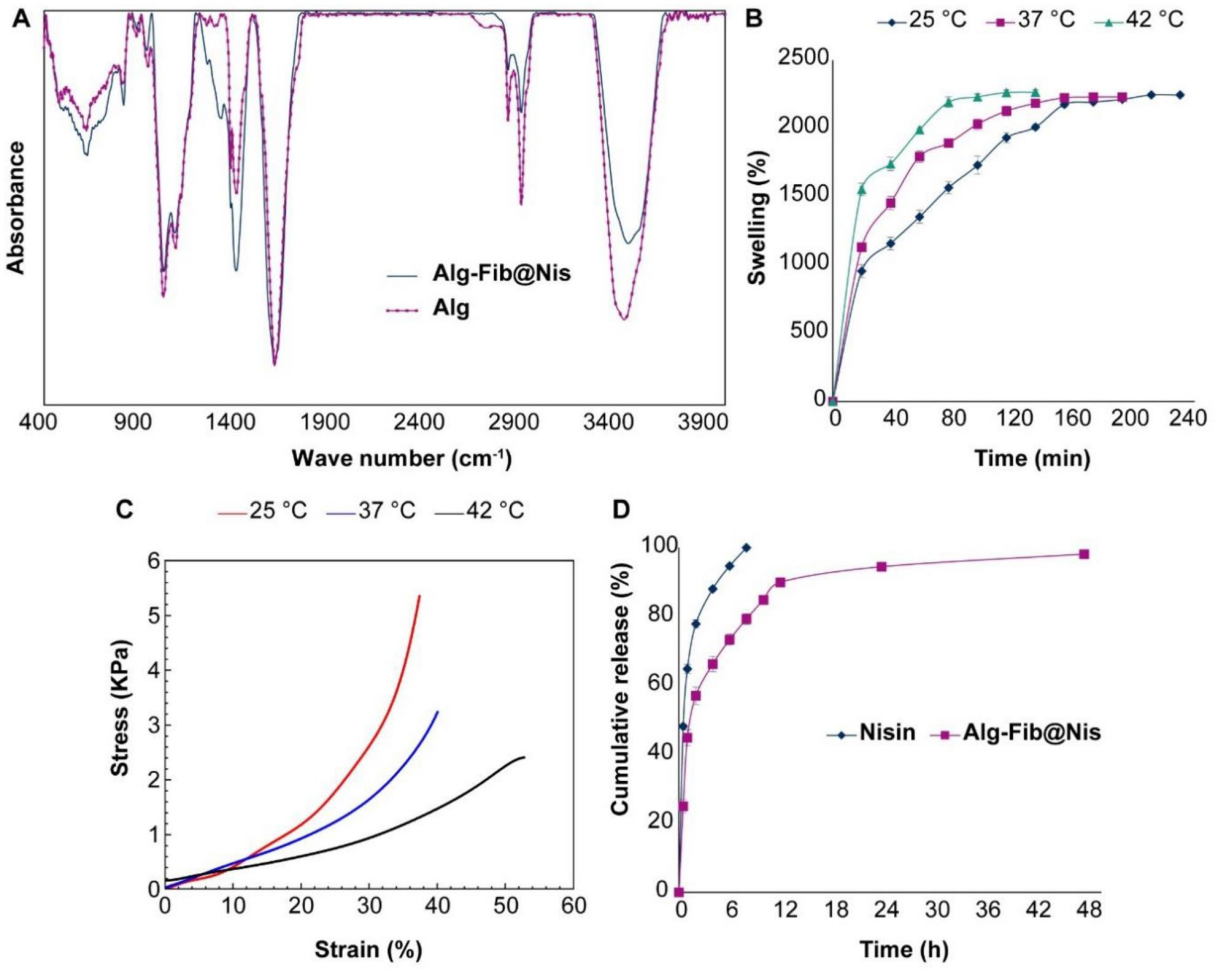
(**A**) FTIR spectra of Alg-Fib@Nis-EDTA hydrogel and alginate polymer. (**B**) The swelling percentages of the Alg-Fib@Nis-EDTA hydrogel over time at various temperatures. The highest swelling percentage was at 240 (2244 ± 13%), 200 (2229 ± 9%), and 140 (2264 ± 17%) min after the incubation at 25, 37 and 42 °C, respectively. Values are mean ± SD, n = 3. SD: standard deviation. (**C**) Stress–strain diagram of the hydrogels after swelling in 25, 37, and 42° C using the extension rate of 5 mm/min. (**D**) Release profile of nisin from Alg-Fib@Nis-EDTA hydrogel compared to free nisin at different times in PBS at 37 °C. Data points are mean ± SD, (n = 3). SD: standard deviation.

Figure 3A shows the FTIR spectra of alginate polymer and Alg-Fib@Nis-EDTA hydrogel. The FTIR spectrum of alginate polymer shows peaks at 3470 and 1029 cm^−1^, related to –OH and –CO groups, respectively. Appearance of peaks at 1620 and 1420 cm^−1^ can be assigned to symmetric and asymmetric tensile vibrations of –COO groups, respectively. Shehzad et. al.^53^ have also reported symmetric and asymmetric tensile vibrations of –COO groups of alginate in corresponding to the peaks of 1630 and 1465 cm^−1^. The FTIR spectrum of Alg–Fib@Nis–EDTA hydrogel also presents all the major peaks of alginate. However, the intensity of the peak at 3470 cm^−1^ decreased due to a reduction of –OH groups upon formation of hydrogel. Furthermore, the intensity of the peaks in the range 1200–1500 cm^−1^ presented an increase, which can be due to the tensile vibration of –CN groups present in fibrinogen and nisin. These results are in accordance with the previous results^54–57^.

#### Swelling ratio

Considering the importance of hydrogels’ swelling in achieving a good system for drug delivery to the wound site and the fact that the exudation of inflammatory fluid and tissue fluid slows down the healing process, a dressing with a high swelling capacity leads to the absorption of excess secretions and rapid wound healing^58^. Therefore, we evaluated the swelling behavior of Alg-Fib@Nis-EDTA at 25, 37, and 42 °C (Fig. 3B). After immersion of the hydrogel in simulated wound fluid (SWF)^59^, the swelling percentage of hydrogels were determined at various time points. According to the results presented in Fig. 3B, increasing temperature did not result in a remarkable difference in the amount of the maximum swelling percentage in 25, 37, and 42 °C (approximately 2200% in the three tested temperatures). However, increasing temperature caused the hydrogel samples reached their maximum swelling in a shorter period of time (120 min for 42 °C compared to 240 min for 25 °C). Salehi et al.^51^ reported 342 ± 18% at 240 min as the maximum swelling of the hydrogel and then the percentage of swelling decreased. They speculated that the interaction between carboxylic acid functional groups of alginate with the surrounding hydrophilic medium can mediate the swelling^51^. Also, in a review article by Ching et al.^60^, it was stated that as the temperature increases, the structure of the gel becomes more open, the size of the pores and the porosity of the gel increase, and as a result, the rigidity of the gel decreases. This is consistent with our findings in the current study because as the temperature rises, the hydrogel reaches its maximum swelling in a shorter time, after which the gel structure begins to open and loses its rigid structure.

#### Mechanical properties

As shown in Fig. 3C, the mechanical properties of the designed hydrogel after swelling in 25, 37, and 42° C decrease with increasing temperature. The highest mechanical stability (5.4 ± 0.1 kPa) of the swolled hydrogel was detected at 25 °C. Increasing temperature to 37 °C decreased mechanical stability (3.2 ± 0.1 kPa). The swollen hydrogel presented the minimum mechanical stability (2.4 ± 0.05 kPa) at 42 °C. It seemed that opening the structure of the hydrogel upon its swelling leads to loosening of the bonds and lower mechanical stability.

#### Encapsulation efficiency and drug release

Nisin encapsulation efficiency was calculated based on Eq. (3) in method section as 93 ± 2.57%. Moreover, the nisin release from Alg-Fib@Nis-EDTA hydrogel was evaluated (Fig. 3D). Based on the results, it took 48 h for nearly all the loaded nisin (98.15%) to be released from the hydrogel. However, the hydrogel presented the highest rate of nisin release during the first 6 h (nearly 50%). Whereas, nearly 100% of the free nisin was released during the same time (6 h) from the dialysis bag to the PBS solution. The initial rapid release of the drug from the hydrogel can be attributed to the nisin molecules interacted weakly via Van der Waals forces and/or hydrogen bonding with the hydrogel moieties or nisin molecules loaded on the superficial parts of the hydrogel, which were released quickly from the hydrogel through diffusion upon swelling of the gel^61–65^. Zohri et al.^66^ have observed an approximately 30% release of nisin from chitosan/alginate nanoparticles during the first hour and 85% release in two hours^66^. On the other hand, Bernela et al.^56^, reported that nearly 60 and 86% of the loaded nisin were released from alginate-chitosan-pluronic nanocomposite during 24 and 240 h, respectively.

#### Cytotoxicity assay

**Figure 4:**
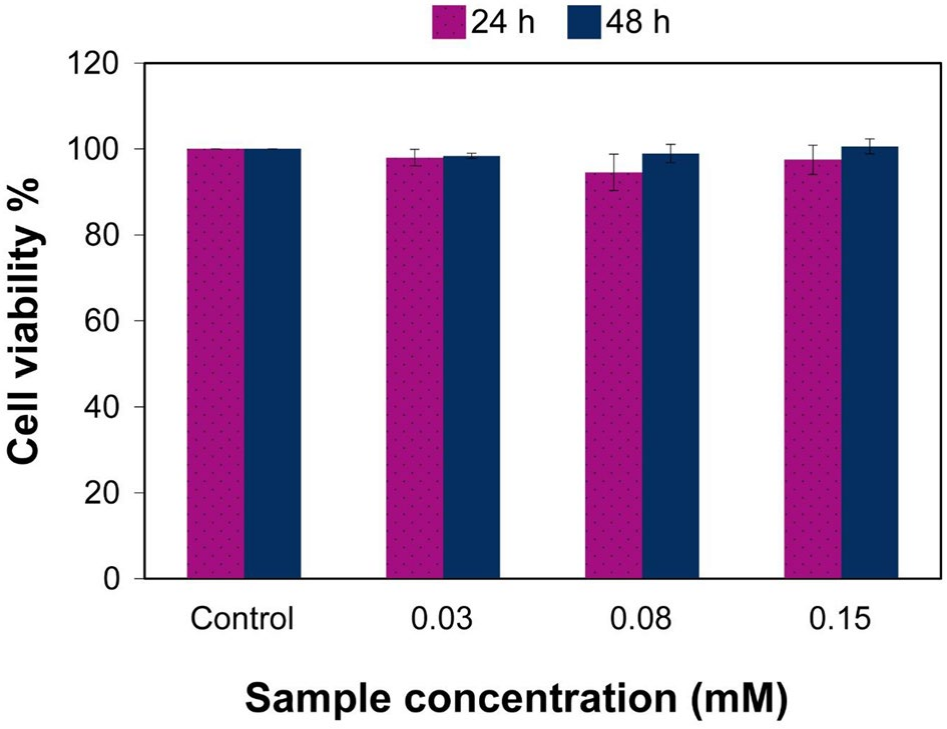
Viability of A431 human epidermis cells by MTT assay after 24 and 48 h post cell treatment with 0.03, 0.08 and 0.15 mM Alg-Fib@Nis-EDTA (nisin based concentrations). Cell viabilities are normalized to the control cells and represent the mean ± SEM. n = 3. SEM: standard error of mean.

The cytotoxicity of Alg-Fib@Nis-EDTA hydrogels loaded with 0.03, 0.08, and 0.15 mM nisin was determined in A431 human epidermis cells at 24 and 48 h using MTT assay (Fig. 4). According to the results, the hydrogels with the tested nisin concentrations did not induce any sign of cytotoxicity in the studied epidermis cell line. The observed cytocompatibility made the Alg-Fib@Nis-EDTA hydrogel suitable for the next step of our in vivo studies in rats.

#### Antibacterial activity

**Figure 5:**
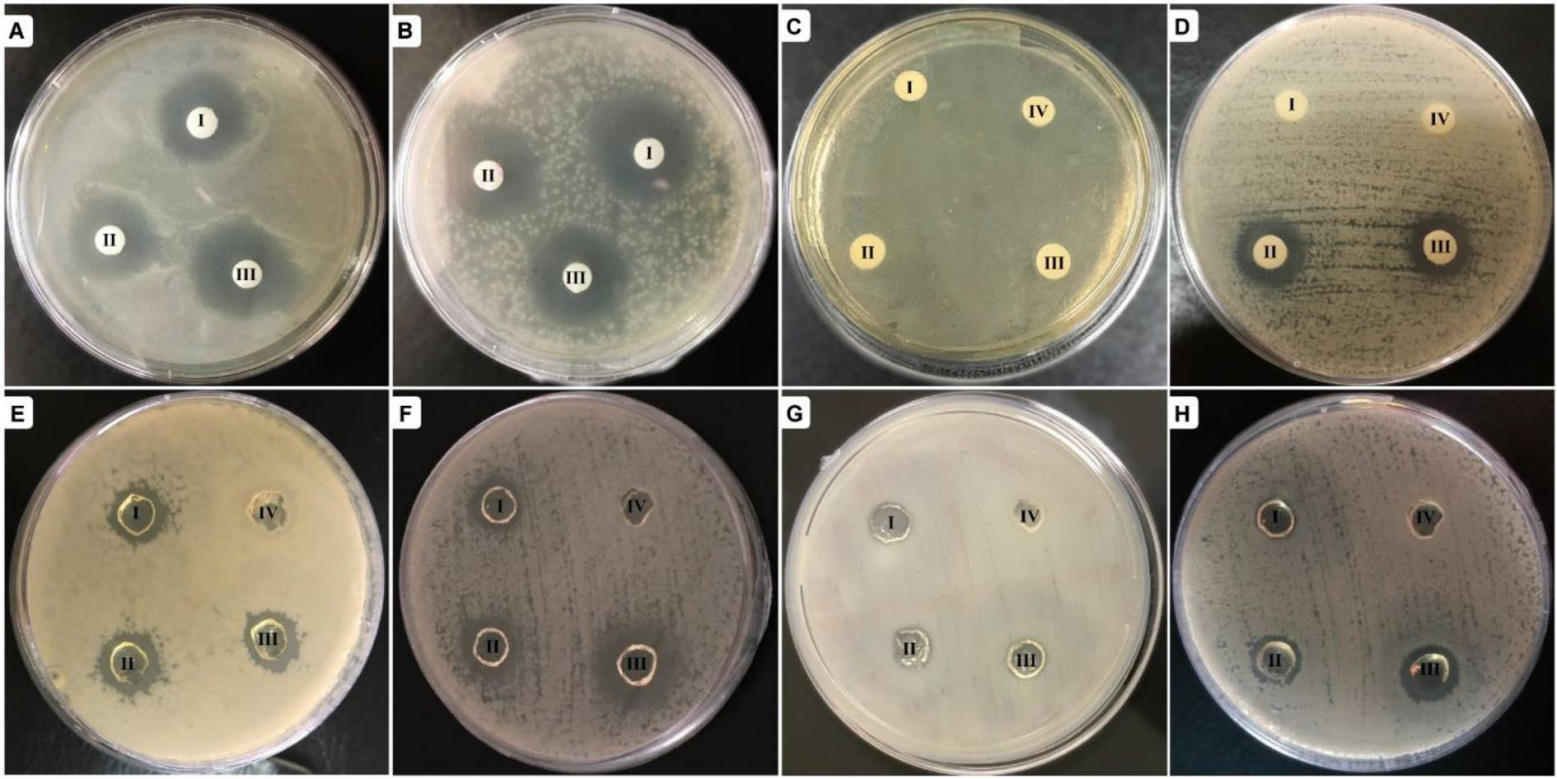
Inhibition zones around the prepared hydrogels. (**A**–**D**) Non-cross-linked and (**E**–**H**) cross-linked hydrogels. The plates in (**A**), (**C**), (**E**), and (**G**) are *Escherichia coli* cultures. The plates in (**B**), (**D**), (**F**), and (**H**) present *Staphylococcus aureus* cultures. In each plate, the hydrogels numbered as I–III contain 0.03, 0.08, and 0.15 mM nisin; number IV represents the control sample. In the plates displayed in (**A**) and (**B**), the controls are avoided due to repetition in the other 6 plates.

Antibacterial effect of Alg-Fib@Nis-EDTA hydrogel with 0.03, 0.08, and 0.15 mM nisin was determined against pathogenic strains of *Escherichia coli* and *Staphylococcus aureus* as representatives of gram-negative and gram-positive bacteria, respectively, according to the procedure mentioned in method *section.* The inhibition zone was observed around non-cross-linked and cross-linked hydrogels (Fig. 5). Growth inhibition zones of *S. aureus* and *E. coli* were measured and presented in Table 1.

**Table 1:** Diameters of inhibition zones caused by different preparations of hydrogels in *S. aureus* and *E. coli* cultures.

Diameter of inhibition zone (mm)				
	Non-cross-linked hydrogel		Cross-linked hydrogel	
	*S. aureus*	*E. coli*	*S. aureus*	*E. coli*
Alg-Fib@Nis-EDTA (0.03 mM)	18.5 ± 0.5	15.9 ± 0.8	13.2 ± 0.4	13.1 ± 0.5
Alg-Fib@Nis-EDTA (0.08 mM)	16.3 ± 0.5	16.8 ± 0.3	14.3 ± 0.6	14.8 ± 0.2
Alg-Fib@Nis-EDTA (0.15 mM)	17.6 ± 1.5	18.3 ± 0.7	15.6 ± 0.8	14.7 ± 0.8
Alg-Fib@Nis (0.03 mM)	0	0	0	0
Alg-Fib@Nis (0.08 mM)	14.6 ± 0.7	0	12.1 ± 0.3	0
Alg-Fib@Nis (0.15 mM)	15.2 ± 0.8	0	13.4 ± 0.5	0
Alg-Fib(Control)	0	0	0	0

Values represent the mean ± SD, n = 3, SD: standard deviation.

The average diameters of inhibition zones in all tested concentrations of nisin for non-cross-linked and cross-linked hydrogels were 17.2 ± 1.06 and 14.3 ± 0.97 mm, respectively. Besides, the hydrogels lacking EDTA (Alg-Fib@Nis) could not inhibit growth of *E. coli*. The Alg-Fib@Nis hydrogel samples containing 0.03 mM nisin could not inhibit growth of *S. aureus*. The inhibition zones were observed in all the tested concentrations of nisin around the Alg-Fib@Nis-EDTA hydrogels for the two bacterial strains with comparable diameters. The comparable inhibition zone in all the tested concentrations indicates that the synthesized hydrogel presents acceptable antibacterial properties^67^. Regarding technical limitations presented by the hydrogel, we could not detect significant differences in growth inhibition imposed by various concentrations of nisin. Therefore, MIC and MBC were determined to obtain more accurate data.

The data obtained from MIC and MBC tests are reported in Table 2. According to our findings, presence of nisin in the studied concentrations in the Alg-Fib@Nis-EDTA hydrogel killed and inhibited the growth of both gram-positive and gram-negative bacteria. As expected, alginate and fibrinogen did not show any antibacterial effect in agreement with Alboofetileh et al.^68^ who reported that alginate had no antibacterial effect on either gram-positive or gram-negative bacteria. In another study, Påhlman et al.^69^ reported a similar lack of antibacterial properties for fibrinogen. Furthermore, EDTA had inhibitory and lethal properties only on gram-negative bacteria such as *E. coli*^70^.

**Table 2:** Minimum inhibitory and bactericidal concentrations (MIC and MBC) (mM) of alginate, fibrinogen, EDTA, nisin, and the prepared hydrogel against gram-positive *Staphylococcus aureus* (ATCC 25923) and gram-negative *Escherichia coli* (ATCC 25922).

	*S. aureus*		*E. coli*	
	MIC	MBC	MIC	MBC
Alginate	+	+	+	+
Fibrinogen	+	+	+	+
EDTA	+	+	−	−
0.03 mM Nisin	+	+	+	+
0.08 mM Nisin	−	+	+	+
0.15 mM Nisin	−	−	+	+
Alg-Fib@Nis-EDTA(0.03 mM)	−	−	−	−
Alg-Fib@Nis-EDTA(0.08 mM)	−	−	−	−
Alg-Fib@Nis-EDTA(0.15 mM)	−	−	−	−

Inhibitory or lethal effect: −; No inhibitory or lethal effect: +.

Moreover, according to our observations and in parallel with the published reports, nisin presented an inhibitory and lethal effect on gram-positive bacteria (*S. aureus*)^71^. Based on MIC and MBC tests, 0.08 mM nisin could inhibit the bacterial growth, and 0.15 mM could kill *S. aureus*. Concentrations less than 0.08 mM (0.03 mM) was not able to inhibit the growth of *S. aureus*. However, 0.03 mM nisin could kill *S. aureus* when applied in the form of the Alg-Fib@Nis hydrogel. Interestingly, the investigated concentrations of nisin incorporated in the Alg-Fib@Nis hydrogel could kill and inhibit the growth of the gram-negative *E. coli*. This amplified potential of nisin in killing both gram-negative and -positive bacteria could be assigned to presence of EDTA in the final hydrogel structure, which can enhance the effect of nisin^25^.

#### Blood coagulation assay

**Figure 6:**
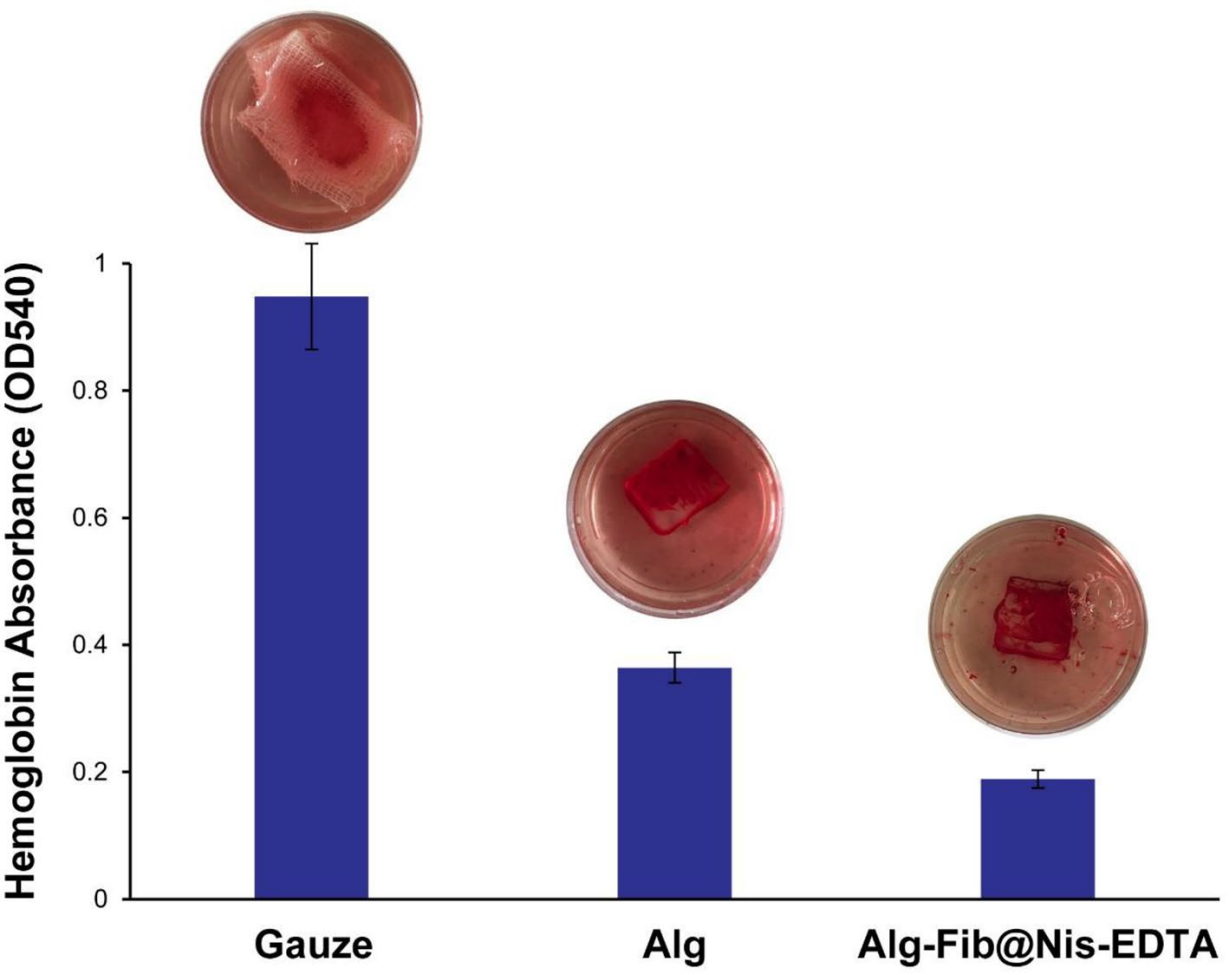
In vitro blood coagulation test. The absorption of the hemoglobin in the rinsing water was measured in 540 nm. The images of the rinsing water from the three samples of gauze dressing, Alg hydrogel, and Alg-Fib@Nis-EDTA hydrogel are displayed to imply the remarkable differences. Data are mean ± SD (n = 3).

In the process of healing skin wounds, especially deep wounds, accelerating the blood clotting process is of special importance. The ability of a dressing to speed up the blood clotting process can be assessed using a blood clotting test^72^. The results of this test are reported in Fig. 6. According to Fig. 6, the absorbance of hemoglobin in the Alg-Fib@Nis-EDTA hydrogel sample is significantly lower than the other two samples containing gauze and Alg hydrogel, which indicates the higher ability of the Alg-Fib@Nis-EDTA hydrogel in blood clot formation. The dishes containing the water used for washing were also displayed to show the remarkable differences between the three samples.

### Animal studies

#### In vivo wound healing test

**Figure 7:**
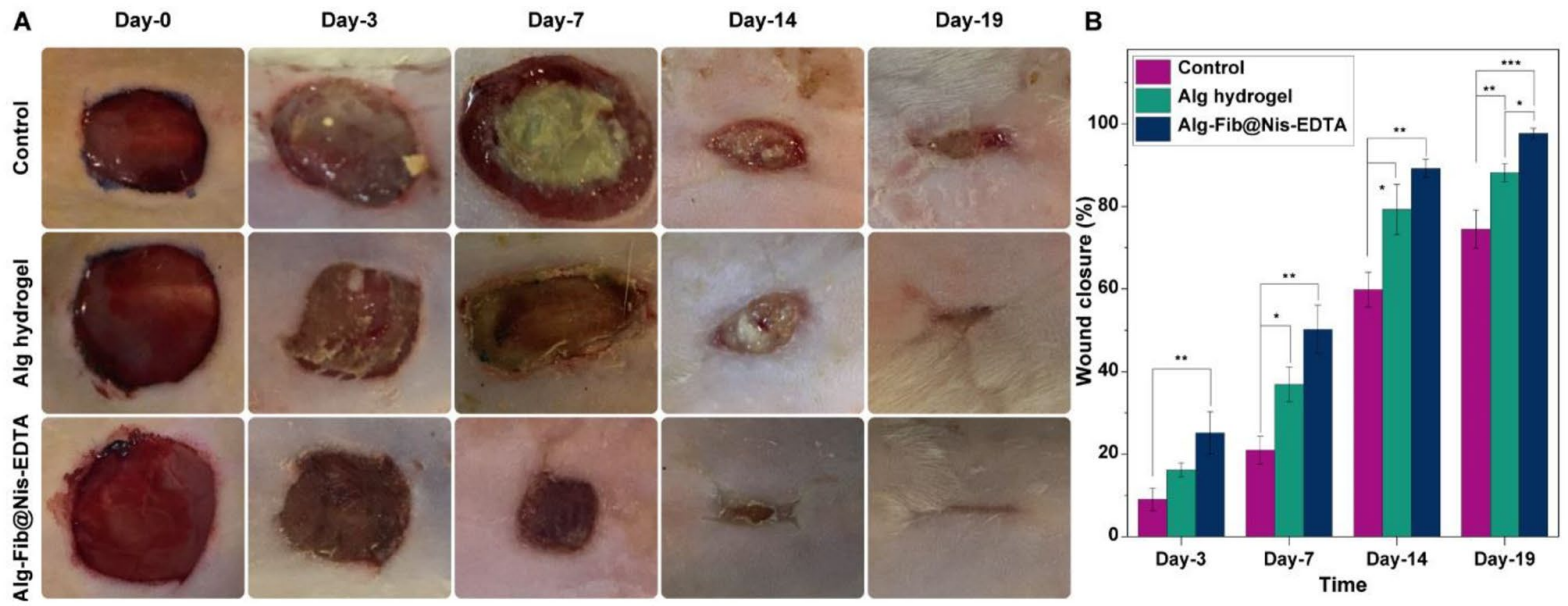
In vivo wound healing results. (**A**) Macroscopic appearances of the wounds treated 1st, 3rd, 7th, 14th and 19th days post-wounding. (**B**) Histogram comparing the wound closure on 1st, 3rd, 7th, 14th, and 19th days post-wounding. Values represent the mean ± SD, n = 3, *p < *0 *.05, **p < 0 .01, and ***p < 0.001. SD: standard deviation.

An animal test was performed to evaluate the effectiveness of the designed hydrogel in skin wound healing. The wound healing process was monitored in the 1st, 3rd, 7th, 14th, and 19th days after the creation of the wound at the specified site. The images and sizes of the wounds are displayed in Fig. 7A. In the group receiving Alg hydrogel dressing, some infection and inflammation were observed and the healing process was relatively slow. The same situation was observed in the control group, which was bandaged only with sterile gauze. While the group treated with Alg-Fib@Nis-EDTA hydrogel did not show any sign of infection and presented improved recovery process compared to the control and Alg hydrogel groups. Moreover, the wound size reduction was calculated using Eq. 5 in order to quantitatively evaluate the wound healing process, which is shown in Fig. 7B. According to the results, the group receiving Alg-Fib@Nis-EDTA hydrogel dressing presented 54.9 ± 13.3% healing on the 7th day, while the control and Alg hydrogel groups presented 8.2 ± 1.7% and 30 ± 3.6%, respectively, at the same time. According to previous studies, healing more than 50% of the wound in 14 days in the hydrogel dressing group indicates proper function of the designed dressing^73–75^.

Also, the wound healing of the Alg-Fib@Nis-EDTA hydrogel group is higher than other groups on 14th day (97.9% ± 0.2). Rezvanian et al.^75^ prepared an Alginate-Pectin hydrogel film loaded with Simvastatin, and reported the percentage of wound closure over the 21-day course of treatment. At last, the hydrogel group containing the Simvastatin reached 99.3 ± 0.5%, while the control group showed a 90 ± 0.0% improvement. However, they observed no improvement until the 7th day of the experiment^75^. While we detected a significant improvement on the 7th day of the experiment.

#### Histopathology

**Figure 8:**
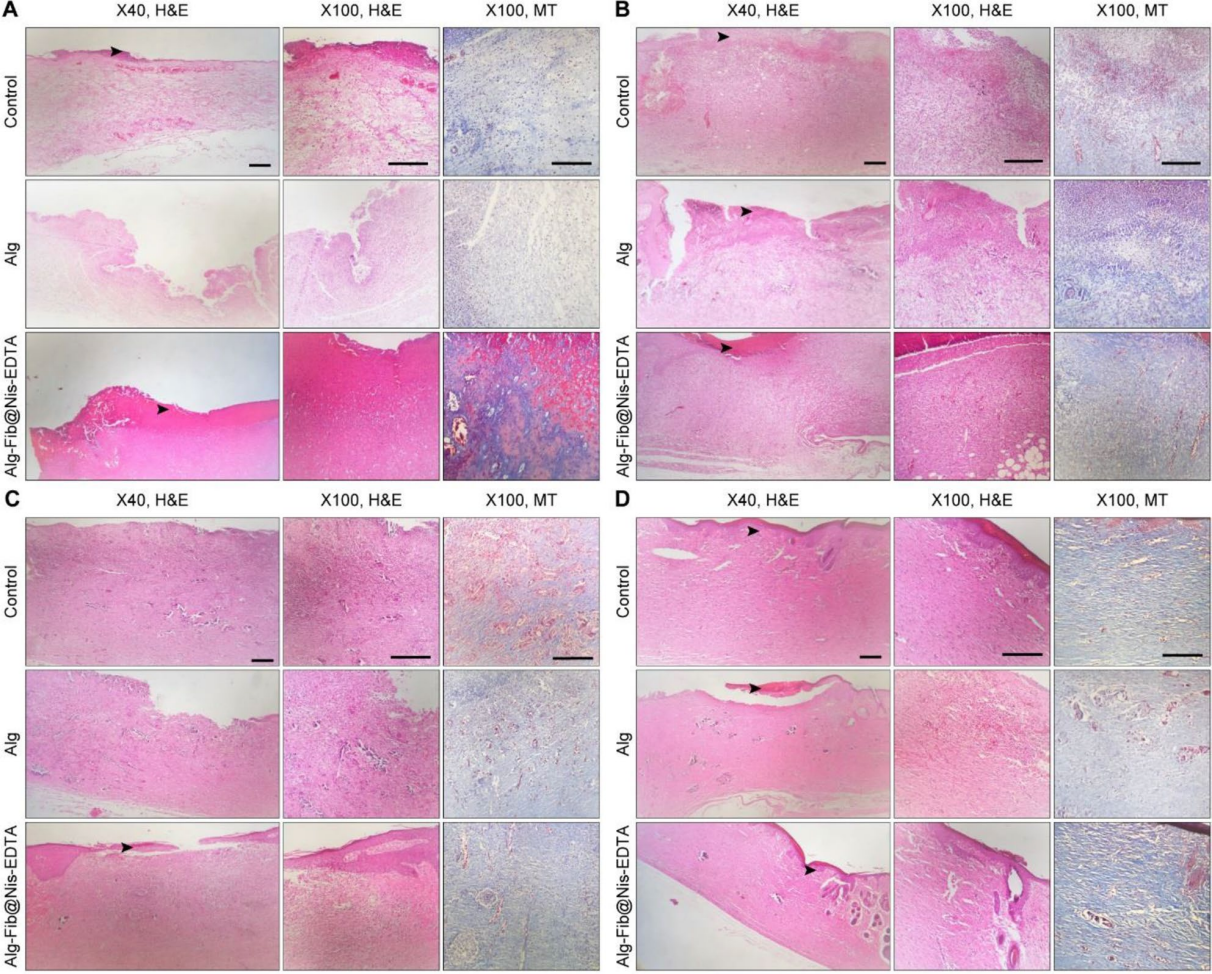
(**A**) H&E and MT stained microscopic sections of healed incisions in the different experimental groups at 3 days’ post-treatment. (**B**) H&E and MT stained microscopic sections of healed incisions in the different experimental groups at 7 days’ post-treatment. (**C**) H&E and MT stained microscopic sections of healed incisions in the different experimental groups at 14 days’ post-treatment. (**D**) H&E and MT stained microscopic sections of healed incisions in the different experimental groups at 19 days’ post-treatment. Black arrows: crusty scab, White arrows: re-epithelialization.

Histopathological studies are needed to evaluate the effectiveness of a dressing. Through these studies, different stages of wound healing can be observed. Histopathological analysis of the skin wounds was performed by H&E and MT staining, as shown in Fig. 8. In the control group, the wounds left without any treatment evaluated on the 3rd, 7th, and 14th days post-treatment showed polymorphonuclear (PMNs) inflammatory cells infiltration and granulation tissue formation. Besides, the wound was covered by a crusty scab. However, the epidermal layer partially regenerated on day 19th. Histopathological evaluation of the Alg hydrogel treatment group on the days 3rd, 7th, 14th, and 19th showed a close resemblance to the control group along with the presence of severe inflammation and granulation tissue formation as seen in Fig. 8.

Micrographs of the Alg-Fib@Nis-EDTA hydrogel treatment group on 3rd and 7th days post-treatment displayed a severe inflammation and a crusty scab covering the wound area (Fig. 8A,B). The epidermal layer has started to regenerate in this group on day 14th as shown in Fig. 8C. Interestingly, the inflammatory response decreased considerably on 19th day post-treatment and a complete epithelial layer was formed (Fig. 8D). This group showed more resemblance to the normal skin, with a thin epidermis and the presence of normal rete ridges.

#### Histomorphometric analysis

**Figure 9:**
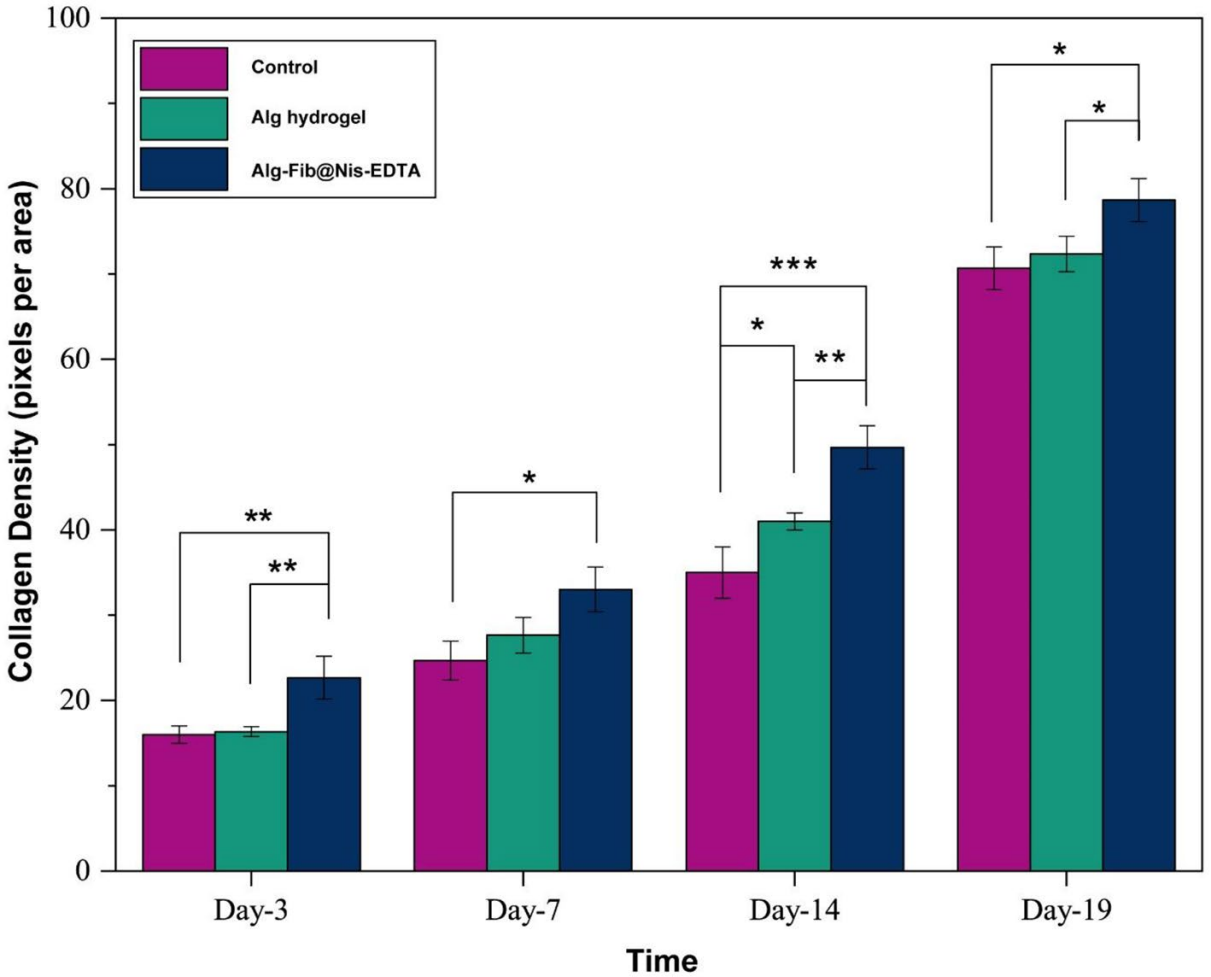
Representative graphical of collagen density at different time-points (3, 7, 14, and 19 d) confirming more wound healing in Alg-Fib@Nis-EDTA compared with other groups. These results were in consistent with the results obtained from wound closure. Values represent the mean ± SD, n = 3, *p* < 0 .05, **p < 0 .01, and **p < 0.001. SD: standard deviation.

The histomorphometric analysis was done on 3, 7, 14, and 19 days after skin injury and the results are summarized in Table 3. Amongst all the groups, re-epithelialization in the Alg hydrogel group was minimum and it was mostly filled with immature granulation tissue. The best re-epithelialization was seen in the group receiving Alg-Fib@Nis-EDTA hydrogel. Using Masson Trichrome staining, the amount of collagen formation in the tissue was examined (Fig. 9). Collagen is one of the most important structural proteins in the body, which is produced by fibroblasts and plays an important role in the process of wound healing and the formation of new tissue. Based on the obtained results, the collagen content was significantly higher in the Alg-Fib@Nis-EDTA hydrogel group compared to Alg hydrogel and control groups. Overall, the healing process in the group receving Alg-Fib@Nis-EDTA hydrogel was more similar to the normal skin and presented the best appearance. Lin et al.^76^ have also reported the acceleration of the granules production and higher collagen production using an alginate based wound dressing.

**Table 3:** Histomorphometric analysis of the groups receiving gauze, Alg hydrogel, and Alg-Fib@Nis-EDTA hydrogel.

Group	Epitheliogenesis score
Control	0 (3 d) 0 (7 d) 1 (14 d) 2 (19 d)
Alg	0 (3 d) 0 (7 d) 1 (14 d) 3 (19 d)
Alg-Fib@Nis-EDTA	0 (3 d) 0 (7 d) 2 (14 d) 4 (19 d)

Control: negative control, Alg: alginate Hydrogel, Alg-Fib@Nis-EDTA: alginate.

+ fibrinogen + Nisin + EDTA Hydrogel.

## Conclusion

In this scenario, an alginate based hydrogel was designed using fibrinogen, EDTA, and nisin for wound healing. In the first step, the interaction between fibrinogen and alginate was studied using spectroscopic techniques to evaluate the possibility of employing fibrinogen in the hydrogel dressing. The obtained results revealed that fibrinogen conformation is minimally affected by the alginate and can be a suitable option for the preparation of the dressing. In the second step, the Alg-Fib@Nis-EDTA hydrogel was prepared and characterized. Various properties of the hydrogel including formation of the desired bonds, antibacterial resistance, swelling and porosity, mechanical resistance, optimal release rate, acceleration of blood coagulation process, and cell cytotoxicity were determined. The designed scaffold absorbed the secretions of the wound environment due to the high swelling capacity. It also presented the gradual release of nisin and presumably EDTA, which inhibited the growth of both gram-positive and gram-negative bacterial species. It did not show any toxicity against the human epidermis cell line and also showed a very good function in accelerating blood coagulation. Based on the in vivo results, the fabricated biocomposite hydrogel could present an appropriate antibacterial effect accompanied by suitable therapeutic efficacy in the chronic wounds through improving the speed of epithelialization and collagen formation processes.

## Supplementary Information

Supplementary Information.
