# Supplementary material for: Designing a new alginate-fibrinogen biomaterial composite hydrogel for wound healing
Source: Sci Rep. 2022 May 4;12:7213. doi: 10.1038/s41598-022-11282-w (PMC9068811; doi:10.1038/s41598-022-11282-w)
Supplement: Supplementary file 1 — Supplementary Information. [file 41598_2022_11282_MOESM1_ESM.docx]

**Designing a new alginate-fibrinogen biomaterial composite hydrogel for wound healing**

Marjan Soleimanpour^a,#^, Samaneh Sadat Mirhaji^a,#^, Samira Jafari^b^*, Hossein Derakhshankhah^b^, Fatemeh Mamashli^a^, Hadi Nedaei^a^, Mohammad Reza Karimi^c^, Hamidreza Motasadizadeh^d^, Yousef Fatahi^d^, Atiyeh Ghasemi^a^, [Maryam Sadat Nezamtaheri](https://www.sciencedirect.com/science/article/pii/S0928493118323336?casa_token=Om_pSw0Zcx0AAAAA:EBhr6TA6yiNONr9NVnrppcmk0s48XyHBzN8RpqmjYJfJgWagLzyUKvtf0NcrpSQS7uhAGQOo#!)^a^, Mohadese Khajezade^e^, Masoumeh Teimouri^f^, Bahram Goliaei^a^, Cédric Delattre^g,h^, Ali Akbar Saboury^a^*

^a^ Institute of Biochemistry and Biophysics, University of Tehran, Mailbox 13145-1384, Tehran, Iran.

^b^ Pharmaceutical Sciences Research Center, Health Institute, Kermanshah University of Medical Sciences, Kermanshah, Iran.

^c^ Polymer Laboratory, School of Chemistry, College of Science, University of Tehran, PO Box 14155 6455, Tehran, Iran.

^d^ Department of Pharmaceutical Nanotechnology, Faculty of Pharmacy, Tehran University of Medical Sciences, Tehran, Iran.

^e^ Department of petroleum microbiology, Academic Center for Education, Culture and Research (ACECR), Shahid Beheshti University, Tehran, Iran.

^f^ Faculty of New sciences and Technologies, University of Tehran, Tehran, Iran.

^g^ Institut Universitaire de France (IUF), 1 rue Descartes, 75005 Paris, France.

^h^ Université Clermont Auvergne, CNRS, Clermont Auvergne INP, Institut Pascal, F-63000 Clermont-Ferrand, France.

^#^ These authors contributed equally to this work.

*Corresponding authors:

Ali Akbar Saboury: saboury@ut.ac.ir

Samira Jafari: samiraa.jafari1362@gmail.com

**Supporting information:** Figure S1 to S5 and Table S1 to S2.

| 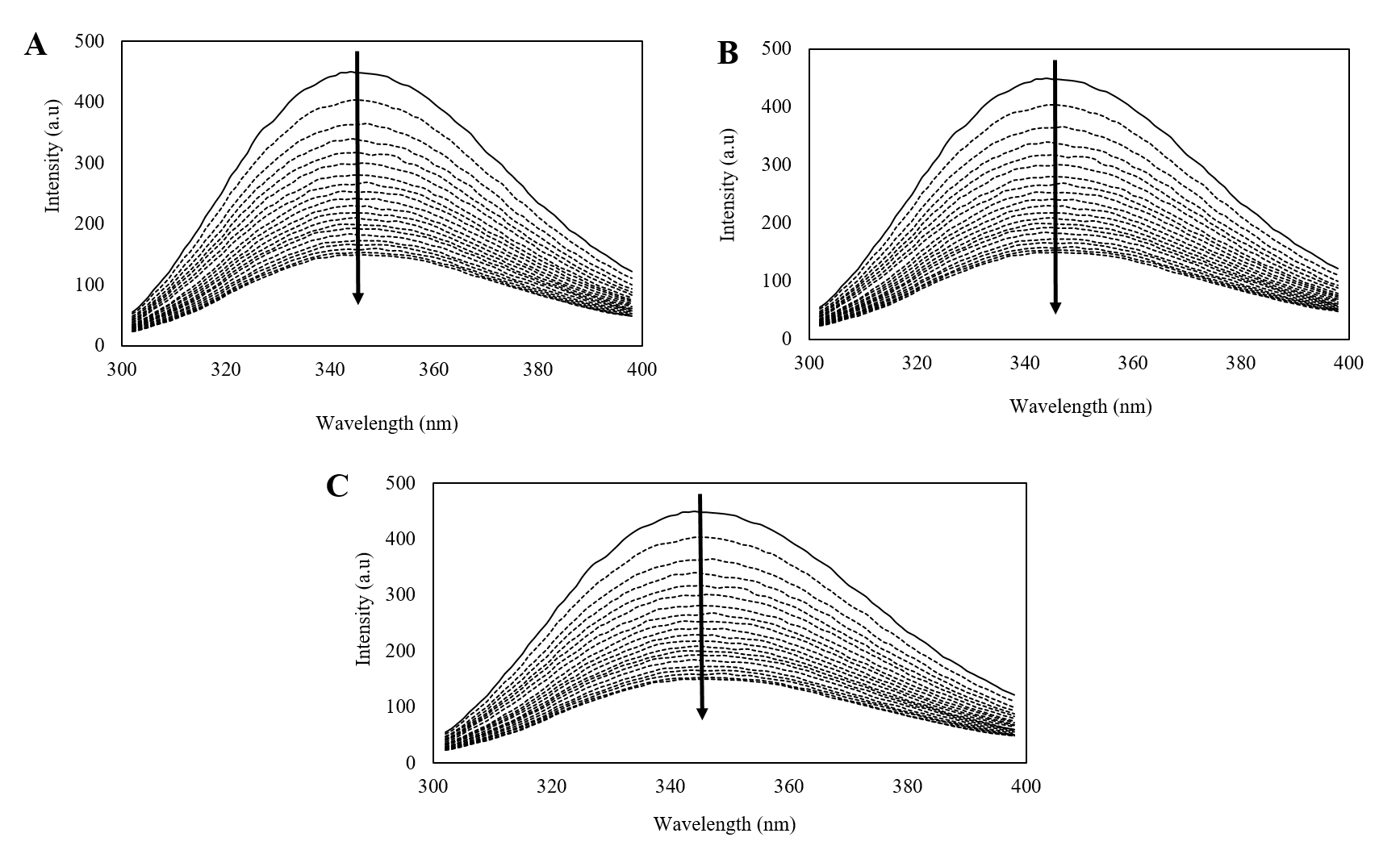 |
| --- |
| **Fig. S1** Intrinsic fluorescence intensity of 1 μΜ fibrinogen in presence of the alginate polymer with molecular weight of 46 kDa in distilled water, pH 7.0 at 25 °C (A), 30 °C (B), and 35 °C (C). The concentration of alginate polymer was between 1.93- 28.98 μΜ. The excitation wavelength was 280 nm. The direction of the arrow shows increasing concentrations of alginate. |

|  |
| --- |
| **Fig. S2** The Stern Volmer plot for quenching fluorescence intensity of 1.0 μM fibrinogen by alginate polymer (46 kDa) at 25, 30, and 35 °C. The excitation and emission wavelengths were 280 nm and 346 nm, respectively. |

|  |
| --- |
| **Fig. S3** The double logarithmic plot of log (F_0_ - F)/F versus log [alginate] at 25, 30, and 35°C. The excitation and emission wavelengths were 280 nm and 346 nm, respectively. |

|  |
| --- |
| **Fig. S4** Far-UV CD spectra of fibrinogen in the absence and presence of 0.4, 0.8, 1.1, and 1.3 μM alginate polymer. The measurements were performed at pH 7.4 and 25 °C. |

|  |
| --- |
| **Fig. S5** Thermal stability of fibrinogen (13.2 μM) in the absence and presence of alginate polymer. The measurements were performed using UV-vis spectroscopy. |

| Table S1. Thermodynamic parameters of the interaction between fibrinogen and alginate polymer (46 kDa) obtained from fluorescence spectroscopy. |
| --- |
| \| ∆S°  (J/K mol) \| ∆H°  (kJ mol^-1^) \| *K*_b_×10^5^  (M^-1^) \| *n* \| ∆G°  (kJ mol^-1^) \| *K*_sv_×10^5^  (M^-1^) \| *T*(°C) \| \| --- \| --- \| --- \| --- \| --- \| --- \| --- \| \| -1163.9 \| -380.5 \| 1 \| 1.3 \| -34.3 \| 0.7 \| 25 \| \| 0.2 \| 0.9 \| -25.9 \| 0.5 \| 30 \| \| 0.07 \| 0.9 \| -22.7 \| 0.2 \| 35 \| |

| **Table S2.** The contents of secondary structure elements of fibrinogen in the absence and the presence of various concentrations of alginate polymer obtained through deconvolution of Far-UV CD data using CDNN software. |
| --- |
| \| 1.3 \| 1.1 \| 0.8 \| 0.4 \| 0 \| [Alg] (μM) \| \| --- \| --- \| --- \| --- \| --- \| --- \| \| 33.4 \| 35.3 \| 30.8 \| 34.1 \| 29.6 \| **Helix (%)** \| \| 11.6 \| 10.6 \| 14 \| 10.7 \| 13.7 \| **Antiparallel (%)** \| \| 5.6 \| 5.5 \| 5.7 \| 5.5 \| 5.7 \| **Parallel (%)** \| \| 18.1 \| 18.1 \| 17.1 \| 18.8 \| 18.6 \| **Beta-turn (%)** \| \| 31 \| 30.3 \| 32.1 \| 30.8 \| 32.4 \| **Random coil (%)** \| |
